# The inhibitors of miR-224-5p, miR-339-5p, and miR-1198-5p improve auditory function by promoting the expression of neuritin in hearing loss mice

**DOI:** 10.1371/journal.pone.0349821

**Published:** 2026-05-21

**Authors:** Pingping Meng, Zhiwei Zhong, Yin Song, Yu Wei, Jiawei Sun, Jingyi Chen, Yunhua Zhang, Jingling Zhu

**Affiliations:** 1 Department of Biochemistry, The Key Laboratory of Xinjiang Endemic & Ethnic Diseases, Shihezi University School of Medicine, Shihezi, Xinjiang, China; 2 Jiaxing Key Laboratory of Pathogenic Microbiology, Jiaxing Center for Disease Control and Prevention, Jiaxing, Zhejiang, China; 3 First Affiliated Hospital of Shihezi University School of Medicine, Shihezi, Xinjiang, China; Sri Venkateswara Veterinary University, INDIA

## Abstract

**Background:**

Sensorineural hearing loss (SNHL) is the main type of hearing impairment, and currently there is a lack of effective preventive or therapeutic drugs. Our previous research revealed that the expression of neurotrophic factor Neuritin decreased in hearing loss. After treatment with recombinant human Neuritin protein, the number of hair cells significantly increased. However, the molecular mechanism underlying the decreased expression of Neuritin in hearing loss remains unclear. Research indicates that Neuritin expression is regulated by microRNAs (miRNAs). This study aims to screen and validate the key miRNAs that regulate the expression of Neuritin and assess the feasibility of these miRNAs as therapeutic targets for SNHL.

**Methods:**

This study utilized a mouse model of SNHL and employed high-throughput sequencing and bioinformatics analysis to screen for miRNAs that regulate the expression of neurofilament proteins in cochlear tissues. Subsequently, in vivo experiments were conducted to verify the regulatory effects of the candidate miRNAs on neurofilament proteins and to evaluate the improvement of SNHL by the corresponding miRNA inhibitors.

**Results:**

After screening and identification, three miRNAs specifically inhibiting the expression of neurofilament protein were obtained, namely miR-224-5p, miR-339-5p and miR-1198-5p. The in vivo experimental results indicated that the inhibitors of the above three miRNAs had a significant improvement effect on the SNHL mouse model.

**Conclusion:**

MiR-224-5p, miR-339-5p and miR-1198-5p can be involved in the pathogenesis of SNHL by regulating the expression of neurofilament proteins. Targeting these miRNAs or their inhibitors can provide new molecular targets and strategies for the prevention and treatment of SNHL.

## Introduction

One of the most common and rapidly growing global public health issues is hearing loss. Approximately 500 million people worldwide suffer from some form of hearing impairment, accounting for over 5% of the world’s population, according to the World Health Organization (WHO) most recent data from 2025 [1]. According to projections, more than 2.5 billion people will have hearing loss by 2050 [2]. Sensorineural hearing loss (SNHL) is the main type of hearing loss, accounting for approximately 90% of all cases of hearing impairment [3,4]. SNHL has multiple major causes, including aging [5,6], genetic mutations [7], exposure to noise [8,9], use of ototoxic medications [10,11], and some chronic systemic disorders [12]. When the hair cells (HCs) of the cochlea, the auditory nerve, or the auditory conduction pathway sustain structural or functional damage, abnormal sound perception and neural signal transmission occur, resulting in a reduction or total loss of auditory sensitivity [13]. Currently, clinical treatment primarily relies on audiologic rehabilitation, such as hearing aids and cochlear implantation. Although these interventions can partially improve auditory function, they exhibit significant limitations. For example, they are unable to achieve structural repair or functional regeneration of damaged auditory cells [14]. Given that mammalian cochlear HCs exhibit limited regenerative capacity following injury or degeneration [15], which results in irreversible hearing loss, preserving the survival and functional viability of existing hair cells becomes essential.

The developmental stages of neurons and HCs are strikingly similar. Accumulating evidence indicates that Neuritin (CPG15, NRN1) is a crucial neurotrophic factor involved in regulating synaptic plasticity and neuronal development, with primary functions including the promotion of neurite outgrowth, maintenance of neuronal survival, and inhibition of neuronal death [16–18]. Previous studies have shown that Neuritin improves synaptic development, stability, and neurotransmitter release in addition to promoting neurogenesis and neural regeneration [19,20]. Additionally, a growing body of research points to its connection to mental and neurological disorders, such as Alzheimer’s disease [21,22], depression [23,24], and memory loss [20]. Neuritin is extensively expressed in normal cochlear tissues, as our earlier research has shown, but after drug-induced injury, its expression is significantly decreased. In addition to preserving neural innervation and reducing drug-induced HCs damage, exogenous delivery of recombinant Neuritin protein boosts the proliferation of supporting cell progenitors and encourages the transdifferentiation of cochlear supporting cells (SCs) into hair cells [25,26]. Nevertheless, little is known about the molecular processes underlying the downregulation of Neuritin expression in the setting of hearing loss. MicroRNAs (miRNAs) have been shown to influence more than one-third of human genes [27]. The growth and maturation of the cochlea are especially influenced by a number of miRNAs, and the onset of hearing problems has been strongly associated with dysregulation of miRNA expression [28,29]. Neuritin, as a neurotrophic factor, is also regulated by miRNA. Therefore, we hypothesize that specific miRNAs may participate in the pathogenesis of SNHL by targeting and regulating Neuritin expression. Furthermore, these miRNAs may serve as potential molecular markers for early diagnosis and targeted therapy of hearing loss.

In this study, we screened the miRNAs that regulate the expression of Neuritin in cochlea of SNHL mice through high-throughput sequencing and bioinformatics analysis. Three miRNAs (miR-224-5p, miR-339-5p, and miR-1198-5p) that specifically inhibited the expression of Neuritin were ultimately obtained through identification. Furthermore, we demonstrated that the inhibitors of miR-224-5p, miR-339-5p, and miR-1198-5p significantly ameliorated SNHL, providing a novel therapeutic strategy for its prevention and treatment.

## Materials and methods

### Animals

Animal experiments were conducted in accordance with the guidelines for the care and use of laboratory animals issued by the National Institutes of Health (NIH) of the United States. The research protocol was approved by the Animal Subject Review Committee of the School of Medicine at Hangzhou Normal University (License Number: HSD-20250909–01). Anesthesia was induced via intraperitoneal injection of a combination of Zoletil 50 and Sumianxin (0.1–0.12 mL/100 g). All drugs used in the animal experiments complied with the standards of animal welfare. The experimental animals were Specific Pathogen Free (SPF) grade CBA mice, purchased from Beijing Vital River Laboratory Animal Technology Co., Ltd., and housed in the SPF animal facility at the Animal Experiment Center of Hangzhou Normal University. The mice were allowed ad libitum access to food and water, and were maintained under controlled environmental conditions, including a 12-hour light/dark cycle, a temperature range of 22–24°C, and a relative humidity of 40–70%.

### Sensorineural hearing loss model

In this study, two models of SNHL were established using a combination of kanamycin sulfate and furosemide (Lasix). One was a high-dose model (kanamycin sulfate 1000 mg/kg + furosemide 500 mg/kg), and the other was a low-dose model (kanamycin sulfate 500 mg/kg + furosemide 100 mg/kg) [30,31]. A total of 45 two-month-old CBA mice with normal hearing were randomly assigned to three groups (n = 15 per group): the normal saline group (control group), the 12-hour drug-induced damage group (high-dose model), and the 24-hour drug-induced damage group (high-dose model). Kanamycin sulfate (0.1 g/ml, 1000 mg/kg) was first administered via subcutaneous injection, followed half an hour later by intraperitoneal injection of furosemide (0.05 g/ml, 500 mg/kg). ABR testing was conducted at 12 and 24 hours post-drug administration to evaluate hearing thresholds. Mice exhibiting a threshold shift of 70–90 dB were selected as experimental subjects, while those showing no significant change or only minor variations in hearing thresholds were excluded from the study.

### ABR testing

ABR thresholds were measured using an auditory evoked potential system (TDT, USA) in a standard sound-attenuated chamber. Mice were fully anesthetized and placed on a heating pad inside the chamber. The ground electrode and recording electrode were inserted subcutaneously into both auricles, while the reference electrode was inserted subcutaneously into the calvarium. Impedance was monitored, and the measurement was initiated when the impedance value was below 3 kΩ. The SigGen RP software was then launched. Click bursts were used as stimuli at a rate of 20 clicks per second. The recording time window was set to 10 ms, with a filter range of 100–3000 Hz, and 1024 sweeps were averaged. Stimulus intensities ranged from 90 to 10 dB SPL, decreasing in 5 dB SPL steps until the characteristic waveform disappeared [32]. The sound intensity at which this waveform (wave II in CBA mice) disappeared was recorded as the hearing threshold for each mouse. The normal hearing threshold for mice was defined as less than 30 dB SPL. The same ABR testing protocol was applied to all groups.

### Screening of candidate miRNAs

Following anesthesia, mice were euthanized via cervical dislocation. The left and right cochleae were carefully excised, and the cochlear bony shells along with surrounding tissues were removed. Under a dissecting microscope, the lateral bony structures of the cochleae were rapidly dissected to isolate the Organ of Corti (n = 6). The collected tissues were flash-frozen in liquid nitrogen and ground into fine powder, followed by total RNA extraction using the TRIzol method (Thermo Fisher Scientific, Waltham, MA, USA). RNA quality was assessed using an Agilent 2100 Bioanalyzer, and samples with poor RNA integrity were excluded from further analysis. High-quality RNA samples were subjected to small RNA sequencing (RNA-seq) at Shenzhen BGI Genomics Technology Co., Ltd., with a sequencing depth of 20 million reads per sample. Differentially expressed miRNAs were identified using DEG-seq analysis, with screening criteria of |log2(fold change)| > 1 and a Q-value < 0.001.

Seven bioinformatics prediction databases—TargetScan, miRTarBase, miRDB, DIANA-TarBase, miRNAMap, miRWalk, and miRMap—were employed to identify potential microRNAs targeting Neuritin (gene symbol: NRN1). The predicted miRNAs were cross-referenced with those identified as differentially expressed in the RNA-seq data. Candidate miRNAs were selected based on the overlap between bioinformatics predictions and sequencing results, specifically focusing on miRNAs that were upregulated in the context of hearing loss and computationally predicted to target Neuritin.

### Quantitative real-time polymerase chain reaction (qRT-PCR)

To detect miRNAs expression, total RNA was extracted from the organ of Corti using TRIzol reagent (Thermo Fisher Scientific, Waltham, MA, USA). RNA samples were reverse-transcribed using the PrimeScript™ RT Kit (Takara Bio, Shiga, Japan). All primers used in this study were synthesized by RiboBio Co., Ltd. (Guangzhou, China, Table 1). QRT-PCR was performed using TB Green® Premix Ex Taq™ II (Takara Bio) on a real-time PCR detection system (Anterium 870wer 3G, Germany). The reaction volume was 10 μL. The PCR conditions were as follows: initial denaturation at 95°C for 10 minutes, followed by 40 cycles of denaturation at 95°C for 5 seconds, annealing at 60°C for 30 seconds, and extension at 70°C for 10 seconds. Each sample was analyzed three times.

**Table 1:** qRT-PCR Primer Sequences.

Primer Name	Sequence
mmu-miR-1198-5p-RT	GTCGTATCCAGTGCAGGGTCCGAGGTATTCGCACTGGATACGACCCAAGC
mmu-miR-1198-5p-F	CGCGTATGTGTTCCTGGCTG
miR-R	AGTGCAGGGTCCGAGGTATT
mmu-miR-339-5p-RT	GTCGTATCCAGTGCAGGGTCCGAGGTATTCGCACTGGATACGACCGTGAG
mmu-miR-339-5p-F	CGTCCCTGTCCTCCAGGAG
mmu-miR-93-3p-RT	GTCGTATCCAGTGCAGGGTCCGAGGTATTCGCACTGGATACGACCGGGAA
mmu-miR-93-3p-F	CGCGACTGCTGAGCTAGCAC
mmu-miR-145a-5p-RT	GTCGTATCCAGTGCAGGGTCCGAGGTATTCGCACTGGATACGACAGGGAT
mmu-miR-145a-5p-F	CGGTCCAGTTTTCCCAGGA
mmu-miR-1247-5p-RT	GTCGTATCCAGTGCAGGGTCCGAGGTATTCGCACTGGATACGACTCCGGG
mmu-miR-1247-5p-F	GACCCGTCCCGTTCGTC
mmu-miR-133a-3p-RT	GTCGTATCCAGTGCAGGGTCCGAGGTATTCGCACTGGATACGACCAGCTG
mmu-miR-133a-3p-F	GCGTTTGGTCCCCTTCAAC
mmu-miR-224-5p-RT	GTCGTATCCAGTGCAGGGTCCGAGGTATTCGCACTGGATACGACAACGGA
mmu-miR-224-5p-F	CGCGCGTAAGTCACTAGTGGT
mmu-miR-181a-2-3p-RT	GTCGTATCCAGTGCAGGGTCCGAGGTATTCGCACTGGATACGACGGTACA
mmu-miR-181a-2-3p-F	CGACCACCGACCGTTGAC
mmu-miR-1247-3p-RT	GTCGTATCCAGTGCAGGGTCCGAGGTATTCGCACTGGATACGACGCTCCA
mmu-miR-1247-3p-F	CGCGGGAACGTCGAGAC
mmu-miR-21a-3p-RT	GTCGTATCCAGTGCAGGGTCCGAGGTATTCGCACTGGATACGACGACAGC
mmu-miR-21a-3p-F	CGCAACAGCAGTCGATGG
mmu-miR-133b-3p-RT	GTCGTATCCAGTGCAGGGTCCGAGGTATTCGCACTGGATACGACTAGCTG
mmu-miR-133b-3p-F	GCGTTTGGTCCCCTTCAAC
mmu-miR-3073a-5p-RT	GTCGTATCCAGTGCAGGGTCCGAGGTATTCGCACTGGATACGACGGCTGG
mmu-miR-3073a-5p-F	CGGTGGTCACAGTTGGCG
mmu-miR-196a-5p-RT	GTCGTATCCAGTGCAGGGTCCGAGGTATTCGCACTGGATACGACCCCAAC
mmu-miR-196a-5p-F	CGCGCGTAGGTAGTTTCATGTT
mmu-miR-211-5p-RT	GTCGTATCCAGTGCAGGGTCCGAGGTATTCGCACTGGATACGACAGGCAA
mmu-miR-211-5p-F	CGCGTTCCCTTTGTCATCCT
mmu-miR-6395-RT	GTCGTATCCAGTGCAGGGTCCGAGGTATTCGCACTGGATACGACTAAACA
mmu-miR-6395-F	GCTGGCCCTCTCTGCCC
MUS-U6-F	CGCTTCGGCAGCACATATAC
MUS-U6-R	CACGAATTTGCGTGTCATCC

### 293T cell culture and transfection

293T cells (2 × 10⁴) were seeded into 6-cm culture dishes and cultured until they reached 60–70% confluence. Candidate miRNA mimics (sequences provided in Table 2) and the corresponding negative control (mimic NC) were transfected into the cells using Lip3000 Reagent (Guangzhou RiboBio Co., Ltd., Guangzhou, China). Following 24 hours of transfection, the transfected cells were harvested to evaluate the effect of miRNA mimic overexpression on neuritin expression.

**Table 2:** Sequence of Candidate miRNA Mimics.

Primer Name	Sequence
mmu-miR-211-5p	UUCCCUUUGUCAUCCUUUGCCU GCAAAGGAUGACAAAGGGAAUU
mmu-miR-6395	CUGGCCCUCUCUGCCCUGUUUA AACAGGGCAGAGAGGGCCAGUU
mmu-miR-181a-2-3p	ACCACCGACCGUUGACUGUACC UACAGUCAACGGUCGGUGGUUU
mmu-miR-1247-3p	CGGGAACGUCGAGACUGGAGC UCCAGUCUCGACGUUCCCGUU
mmu-miR-3073a-5p	GUGGUCACAGUUGGCGCCAGCC CUGGCGCCAACUGUGACCACUU
mmu-miR-339-5p	UCCCUGUCCUCCAGGAGCUCACG UGAGCUCCUGGAGGACAGGGAUU
mmu-miR-93-3p	ACUGCUGAGCUAGCACUUCCCG GGAAGUGCUAGCUCAGCAGUUU
mmu-miR-1198-5p	UAUGUGUUCCUGGCUGGCUUGG AAGCCAGCCAGGAACACAUAUU
mmu-miR-1247-5p	ACCCGUCCCGUUCGUCCCCGGA CGGGGACGAACGGGACGGGUUU
mmu-miR-21a-3p	CAACAGCAGUCGAUGGGCUGUC CAGCCCAUCGACUGCUGUUGUU
mmu-miR-224-5p	UAAGUCACUAGUGGUUCCGUU CGGAACCACUAGUGACUUAUU
mmu-miR-145a-5p	GUCCAGUUUUCCCAGGAAUCCCU GGAUUCCUGGGAAAACUGGACUU
Negative control	UUCUUCGAACGUGUCACGUTT ACGUGACACGUUCGGAGAATT

### Luciferase reporter assay

The direct interaction between miR-145a-5p, miR-224-5p, miR-339-5p, miR-1198-5p and Neuritin was confirmed using a dual-luciferase reporter assay (Luciferase Reporter Gene Sequence provided in Table 3). The wild-type neuritin sequence and its mutant variant were cloned into the GP-miRGLO vector (empty vector: Control group; wild-type neuritin: WT group; mutated neuritin: MUT group) and co-transfected with miRNA mimics into 293T cells. Following 24 hours of transfection, luciferase activity was measured using a GloMax 20/20 luminometer (Promega, Madison, WI, USA).

**Table 3:** Luciferase Reporter Gene Sequence.

Primer Name	Sequence
NRN1-mmu-miR-1198-5p WT	CTTTATTTTCCTTCCTCTCTTGTTTTAGCTGTTACACATACAGTAATACCTGAATATCCAAC
NRN1-mmu-miR-1198-5p MUT	CTTTATTTTCCTTCCTCTCTTGTTTTAGCTGTTTGTGTAACAGTAATACCTGAATATCCAAC
NRN1-mmu-miR-224-5p WT	GAGAGGGAAAAGGAGAAGGCCAGGGGAATGACTTCAAGAGTGGTGTCCACGTGGGAATCA
NRN1-mmu-miR-224-5p MUT	GAGAGGGAAAAGGAGAAGGCCAGGGGAAACTGAACAAGAGTGGTGTCCACGTGGGAATCA
NRN1-mmu-miR-339-5p WT	GCGACAGCATGGCCAACTACCCGCAGGGCCTGGACGACAAGACGAACATCAAGACCGTGT
NRN1-mmu-miR-339-5p MUT	GCGACAGCATGGCCAACTACCCGCAGGGGGACCTCGACAAGACGAACATCAAGACCGTGT
NRN1-mmu-miR-145a-5p WT	CCACAGCUGCACGGUCACAGCUCUUACGGAUUGCCAGGAAGGGGCGAAAGAUAUGUGGGA
NRN1-mmu-miR-145a-5p MUT	CCACAGCUGCACGGUCACAGCUCUUACCCTAAGCCAGGAAGGGGCGAAAGAUAUGUGGGA

### Basilar membrane explant culture and pharmacological intervention

Postnatal day 3 (P3) neonatal mice were euthanized by decapitation, and the heads were collected into ice‑cold D‑Hanks dissection solution in a laminar flow cabinet. The skin was incised along the midline of the skull using dissection scissors, and the cranium was opened through the foramen magnum. After removal of the brain tissue, both temporal bones were harvested and transferred into ice‑cold D‑Hanks solution. Under a dissecting microscope, the temporal bones were dissected to expose the cartilaginous surface, and the entire inner ear labyrinth was isolated. The cochlear shell was carefully separated from the spiral ligament using forceps and removed. Subsequently, the cochlear epithelium together with the lateral spiral ligament and stria vascularis was completely detached from the modiolus. The tissues connected to the cochlear epithelium were dissected away to obtain an intact basilar membrane. The basilar membrane was then placed on a cell culture insert and cultured in an incubator at 37 °C with 5% CO₂. All reagents were obtained from Sigma-Aldrich Inc. To avoid potential confounding effects of serum-derived growth factors or hormones, all experiments were performed using serum-free medium. Twenty-four hours after plating, explants were subjected to treatment. Control cultures received 0.1% dimethyl sulfoxide (DMSO) in physiological saline, while the experimental group was treated with a mixture of miRNA inhibitors targeting miR-339-5p, miR-1198-5p, and miR-224-5p (1:1:1 ratio, 100 nmol per well; sequences provided in Table 4). The solution was gently mixed and returned to the incubator under standard culture conditions (37 °C, 5% CO₂). After 24 hours of incubation, gentamicin (3 mmol per well) was added to each culture dish, the solution was mixed thoroughly, and cultures were further incubated for an additional 48 hours. Subsequently, the gel droplets containing the explants were collected for immunofluorescence analysis.

**Table 4:** Synthesis Sequences of miRNA Antagomirs (AMs).

Primer Name	Sequence
mmu-miR-339-5p	CGUGAGCUCCUGGAGGACAGGGA
mmu-miR-1198-5p	CCAAGCCAGCCAGGAACACAUA
mmu-miR-224-5p	AACGGAACCACUAGUGACUUA
MircoRNA inhibitor N.C	CAGUACUUUUGUGUAGUACAA

### Drug delivery through the round window of the cochlea

CBA mice were utilized for miRNA antisense oligonucleotide (AM) intervention (sequences provided in Table 5). Following anesthesia induction, the mice were placed on a thermoregulated heating pad to maintain a core body temperature of 38 ± 0.5 °C. A retroauricular surgical approach was performed to access the cochlear bulla, where a 2-mm fenestration was created to expose the round window niche. The left ear received 2 μL of AMs solution containing miR-339-5p, miR-1198-5p, and miR-224-5p at a 1:1:1 ratio (1 mg/mL in sterile normal saline) adsorbed onto a gelatin sponge pledget. The right ear served as a control and received a saline-soaked gelatin sponge via the round window delivery route. Normal saline was used as a negative control to distinguish the specific effects of the AMs intervention. ABR measurements were performed at the following time points: before surgery and on days 0,1, 3, 7, and 14 post-injection. The bulla fenestration was sealed with muscle tissue, and the skin incision was closed using absorbable sutures.

**Table 5:** Synthesis Sequences of miRNA Inhibitor.

Primer Name	Sequence
mmu-miR-339-5p	CGUGAGCUCCUGGAGGAGGACAGGGA
mmu-miR-1198-5p	CCAAGCCAGCCAGGAACACAUA
mmu-miR-224-5p	AACGGAACCACUAGUGACUUA
MircoRNA inhibitor N.C	CAGUACUUUUGUGUAGUACAA

### Western blot

Cells were lysed in RIPA lysis buffer (Beyotime Biotechnology Co., Ltd. Shanghai, China) supplemented with a 1% protease inhibitor cocktail. Following centrifugation at 14,000 rpm at 4 °C for 10 minutes, the supernatant was collected. Total protein concentration was quantified using the bicinchoninic acid (BCA) protein assay kit (Beyotime Biotechnology Co., Ltd.Shanghai, China). Equal amounts of protein were separated by 12.5% sodium dodecyl sulfate-polyacrylamide gel electrophoresis (SDS-PAGE) and subsequently transferred onto a polyvinylidene difluoride (PVDF) membrane at 23 V for 43 minutes. The membrane was blocked with 5% non-fat dry milk in Tris-buffered saline containing Tween 20 (TBST) at room temperature for 2 hours, followed by overnight incubation at 4 °C with rabbit anti-Neuritin monoclonal antibody (1:1000; ab64186, Abcam). The following day, the membrane was incubated with horseradish peroxidase-conjugated goat anti-rabbit IgG secondary antibody (1:10,000; ZB-2301, Zsgb-Bio) at room temperature for 2 hours. Protein signals were visualized using Clarity Western ECL substrate (Bio-Rad Laboratories, Inc.). β-actin (ZSGB-Bio) served as the endogenous loading control. Use the Adobe Photoshop CC 2015 software for grayscale scanning.

### Immunofluorescence

The tissues were fixed in 4% paraformaldehyde (PFA; pH 7.4) at 4°C for 24–48 hours, and then dehydrated in 0.25 M Ethylenediaminetetraacetic Acid (EDTA) (pH 8.0) at 4°C for one week. Basement membrane sections were mounted on slides and blocked with 10% horse serum (Gibco) containing 0.03% saponin in 1 × phosphate buffered saline with 0.1% Triton X-100 for 1 hour at room temperature. Sections were then incubated overnight at 4°C with anti-MyoVIIA antibody(1:500, rabbit monoclonal, Abcam). After washing, the sections were incubated with goat anti-rabbit Alexa Fluor 488 secondary antibody(1:500, Technologies, Paisley, U.K.) for 2 hours at room temperature. Finally, the sections were sealed with 20 μl mounting medium.

### Statistical analysis

There were at least three biological replicates for each condition in every experiment. GraphPad Prism 9 (GraphPad Software, CA, USA) was used to analyze the data, and the results were presented as mean ± standard error of the mean (SEM). Before statistical testing, normality and variance homogeneity were confirmed. Depending on the experimental design, either a one-way or two-way analysis of variance (ANOVA) was used, and then the proper post hoc test for multiple comparisons was conducted. The threshold for statistical significance was set at p < 0.05.

## Results

### Neuritin expression is downregulated in animal models of SNHL

**Fig 1:**
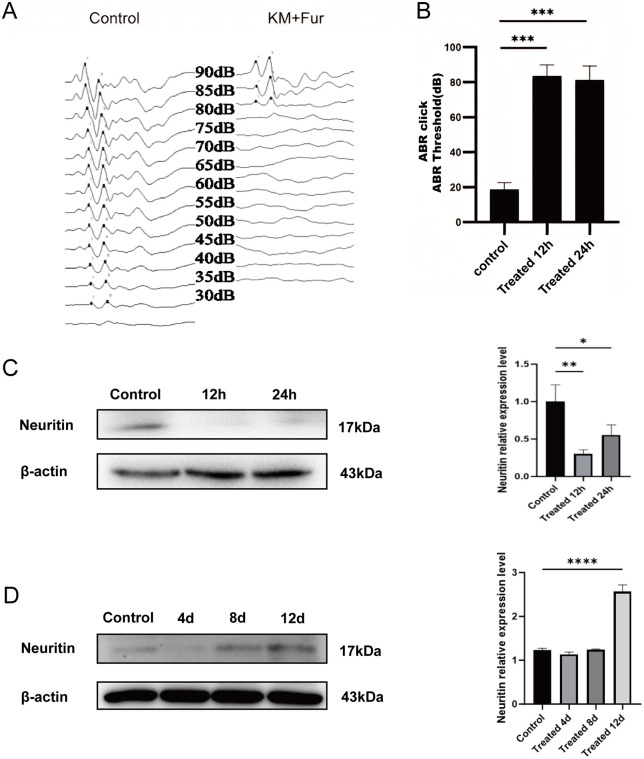
Identification of animal models for sensorineural hearing loss. **A** Changes in hearing thresholds before and after modeling in CBA mice. **B** Statistical analysis of the changes in hearing thresholds. **C-D** Weatern blot detection of changes in Neuritin expression in the cochlea of mice at different time points after modeling. Data are expressed as mean ±SD.*p < 0.05，**p < 0.01，***p < 0.001，****p < 0.0001.

A mouse model of sensorineural hearing loss was successfully established through the combined administration of kanamycin (1000 mg/kg) and furosemide (500 mg/kg). Hearing function assessments demonstrated that control group mice exhibited normal auditory waveforms and maintained hearing thresholds within the normal range. In contrast, significant waveform disturbances were observed at 12 and 24 hours post-model induction (Fig 1A), with hearing thresholds elevated to 70–90 dB (Fig 1B), confirming the successful establishment of the hearing loss model. Further analysis of Neuritin expression in the cochlea following auditory injury revealed a marked decrease at 12 and 24 hours post- induction (Fig 1C), with partial recovery observed starting on day 8 (Fig 1D). These findings indicate a potential association between Neuritin expression levels and hearing loss in mice.

### Identification of miRNAs associated with Neuritin in SNHL

**Fig 2:**
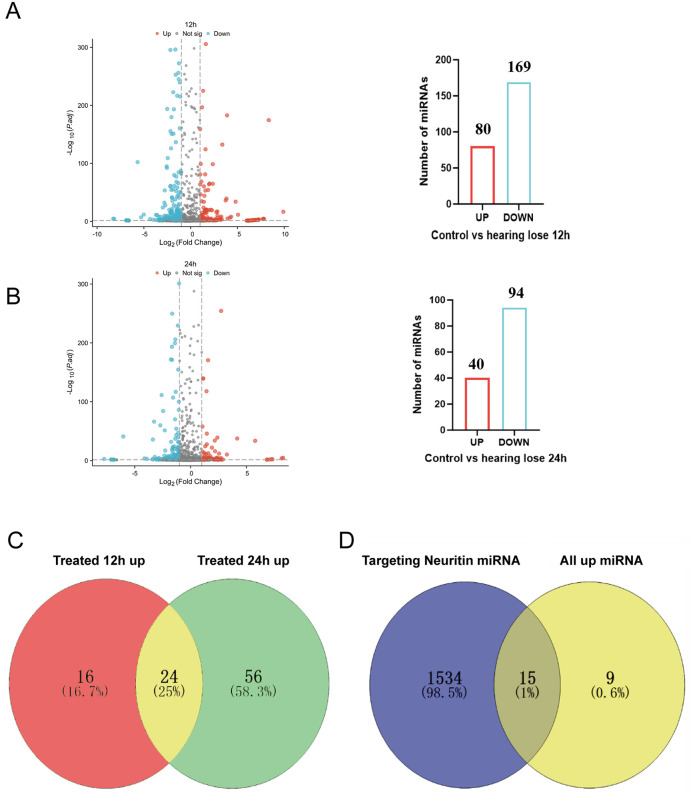
Differential miRNA Analysis and Candidate miRNA Screening. **A** miRNAs with differential expression between the drug injury 12-hour group and the control group. **B** miRNAs with differential expression between the drug injury 24-hour group and the control group.**C** miRNAs that are upregulated in both the 12-hour group and the 24-hour group. **D** The intersection of miRNAs targeting Neuritin and those that are upregulated in both the 12-hour group and the 24-hour group.

RNA was extracted from the Organ of Corti of mice in the control group, the 12-hour model group, and the 24-hour model group and subjected to high-throughput sequencing. Differentially expressed miRNAs in the 12-hour and 24-hour model groups were analyzed using the DEG-seq method, with the screening criteria of |log₂(fold change)| > 1 and q-value < 0.001.. Compared with the control group, 80 miRNAs were significantly upregulated and 169 were significantly downregulated in the 12-hour model group (Fig 2A), whereas 40 miRNAs were upregulated and 94 were downregulated in the 24-hour model group (Fig 2B).

**Fig 3:**
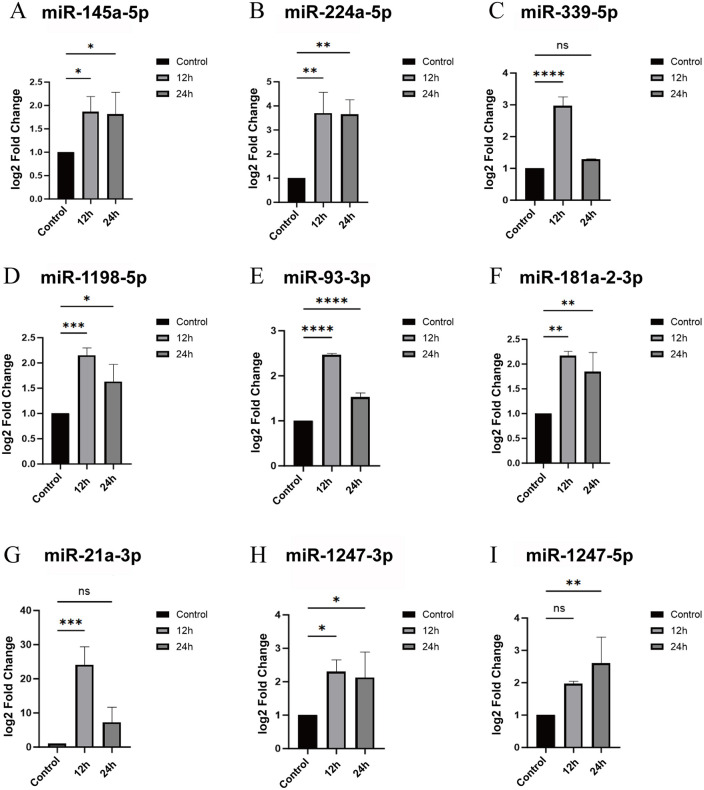
qRT-PCR results of 15 candidate miRNAs. A-I Statistical graph of qRT-PCR results for 15 candidate miRNAs.

Subsequently, bioinformatics approaches were employed to predict miRNAs targeting Neuritin, yielding a total of 1533 candidate molecules. The intersection of these candidates with the 24 significantly upregulated miRNAs (Fig 2C) identified through sequencing (Fig 2D) led to the final selection of 15 miRNAs that are highly expressed in sensorineural hearing loss and potentially target Neuritin (Table 6). To validate the candidate miRNAs identified in the screening, we performed qRT-PCR to assess their expression levels in the Organ of Corti of hearing loss model mice. The results demonstrated that 9 out of the 15 candidate miRNAs were consistently upregulated in both the 12-hour and 24-hour model groups. These miRNAs included miR-21a-3p, miR-93-3p, miR-145a-5p, miR-181a-2-3p, miR-224-5p, miR-339-5p, miR-1198-5p, miR-1247-3p, and miR-1247-5p (Fig 3A–I). Homology analysis show that the sequence homology between humans and mice was above 95% for identified miRNA (Table 7).

**Table 6:** 15 upregulated miRNAs targeting Neuritin in sensorineural hearing loss.

ID	Log2FC (12h)	Log2FC (24h)
mmu-miR-6395	9.902375114	8.294620749
mmu-miR-211-5p	4.883149314	4.180081616
mmu-miR-196a-5p	3.744036623	2.552801226
mmu-miR-3073a-5p	2.17951105	2.387730153
mmu-miR-133b-3p	2.02469942	2.349374161
mmu-miR-21a-3p	1.790449884	2.307428525
mmu-miR-1247-3p	1.751320887	1.916243595
mmu-miR-181a-2-3p	1.620006171	1.56877381
mmu-miR-224-5p	1.618699686	1.460072816
mmu-miR-133a-3p	1.576811863	1.450963418
mmu-miR-1247-5p	1.463477479	1.304519703
mmu-miR-145a-5p	1.308867767	1.243959048
mmu-miR-93-3p	1.209508516	1.132215524
mmu-miR-339-5p	1.142836059	1.116049148
mmu-miR-1198-5p	1.051379351	1.065787458

**Table 7:** Homology of upregulated miRNAs in hearing loss.

ID	Homology
miR-224-5p	95%
miR-339-5p	100%
miR-93-3p	100%
miR-181a-2-3p	95.45%
miR-1247-3p	100%
miR-1247-5p	100%

### Targeted regulation of Neuritin by candidate miRNAs

**Fig 4:**
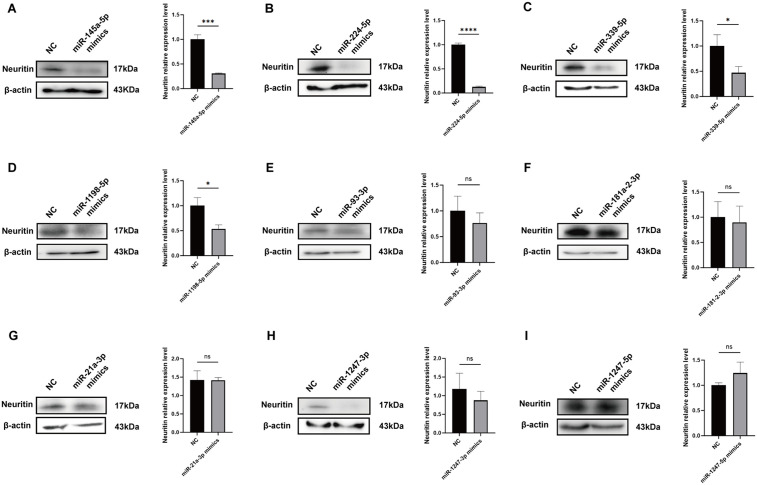
The influence of candidate miRNAs on Neuritin expression. **A-I** Western blot analysis of the effect of candidate miRNAs on Neuritin expression.Data are expressed as mean ±SD.ns *p* > 0.05，*p < 0.05，**p < 0.01, ***p < 0.001, ****p < 0.0001.

To explore the functional relationship between the nine candidate miRNAs and Neuritin, we synthesized miRNA mimics and individually transfected them into 293T cells. The impact of each miRNA on Neuritin protein expression was evaluated by Western blot analysis. The results revealed that transfection with miR-145a-5p, miR-224-5p, miR-339-5p, or miR-1198-5p mimics led to a significant reduction in Neuritin protein levels (Fig 4A–D). In contrast, no notable changes in Neuritin expression were observed in cells transfected with miR-93-3p, miR-181a-2-3p, miR-21a-3p, miR-1247-3p, or miR-1247-5p mimics (Fig 4E–I). These findings demonstrate that miR-145a-5p, miR-224-5p, miR-339-5p, and miR-1198-5p are capable of suppressing Neuritin expression.

**Fig 5:**
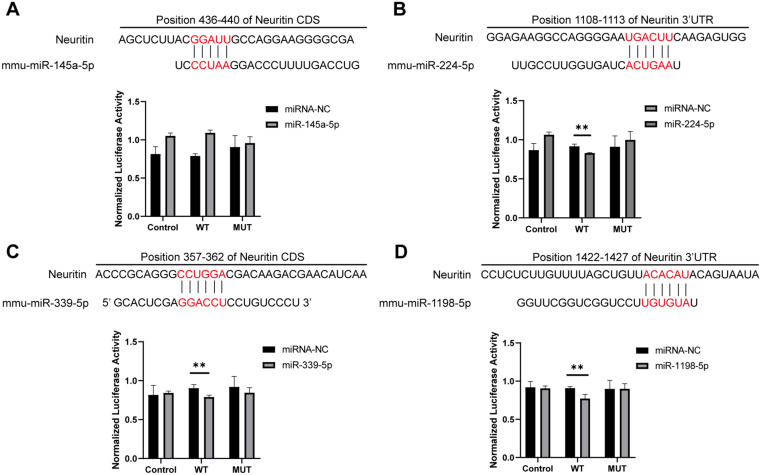
Results of luciferase reporter gene assay. **A-D** Binding sites of candidate miRNAs with Neuritin and quantification graphs of luciferase reporter gene results.

Furthermore, to elucidate the specific binding interactions between Neuritin and four candidate miRNAs (miR-145a-5p, miR-224-5p, miR-339-5p, and miR-1198-5p), we conducted luciferase reporter gene assays. The results demonstrated that, compared to the negative control (NC) group, luciferase activity was significantly decreased in the wild-type (WT) reporter groups co-transfected with miR-224-5p, miR-339-5p, or miR-1198-5p mimics (Fig 5B–D). In contrast, no significant reduction in luciferase activity was observed in the corresponding mutant (MUT) reporter groups compared to the NC group. Notably, in the WT group transfected with miR-145a-5p mimic, luciferase activity was elevated compared to the NC group (Fig 5A), and no significant change was detected in the MUT group, suggesting that miR-145a-5p does not specifically bind to Neuritin. Collectively, these findings indicate that miR-224-5p, miR-339-5p, and miR-1198-5p specifically interact with Neuritin. Therefore, these three miRNAs were selected as the focus for further investigation in this study.

### Effects of miR-339-5p, miR-1198-5p, and miR-224-5p Inhibitors on SNHL

#### Effects of the inhibitor on gentamicin-induced hair cell damage.

**Fig 6:**
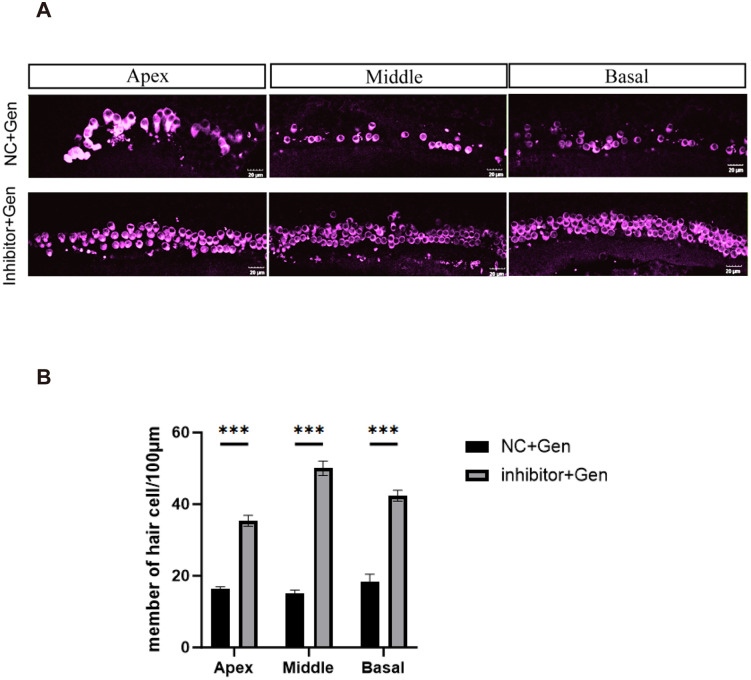
The effect of target miRNA inhibitor on hair cells damaged by gentamicin. **A** The absence of hair cells in the apical, middle and basal lamina regions.**B** Quantitative analysis results of hair cells.***p < 0.001.

HCs are sensory epithelial cells located in the organ of Corti within the inner ear, responsible for transducing sound wave stimuli into electrical signals. These signals are transmitted to spiral ganglion neurons (SGNs) and subsequently relayed via the auditory nerve to brainstem nuclei for auditory processing. Therefore, preserving an adequate number of HCs and their neural innervation is essential for proper auditory function. In this study, the basilar membrane from the cochlea of 3-day-old CBA mice was cultured in vitro. Following 24 hours of initial culture, treatments were administered as follows: the experimental group received a mixture of miR-339-5p, miR-1198-5p, and miR-224-5p inhibitors at a total amount of 100 pmol per well (1:1:1 ratio), whereas the negative control (NC) group received an equal volume of control inhibitor. Immunofluorescence staining revealed that in the NC group, outer hair cells in the apical, middle, and basal turns exhibited a marked reduction in number. In contrast, the miRNA inhibitor-treated group showed minimal loss, with hair cell counts largely preserved across all cochlear regions (Fig 6A). Statistical analysis confirmed a significant difference between the two groups (Fig 6B). These findings indicate that inhibition of miR-224-5p, miR-339-5p, and miR-1198-5p effectively mitigates gentamicin-induced hair cell damage, suggesting that these miRNA inhibitors confer protective effects on basilar membrane hair cells.

### Effects of AM on auditory function in mice with SNHL

**Fig 7:**
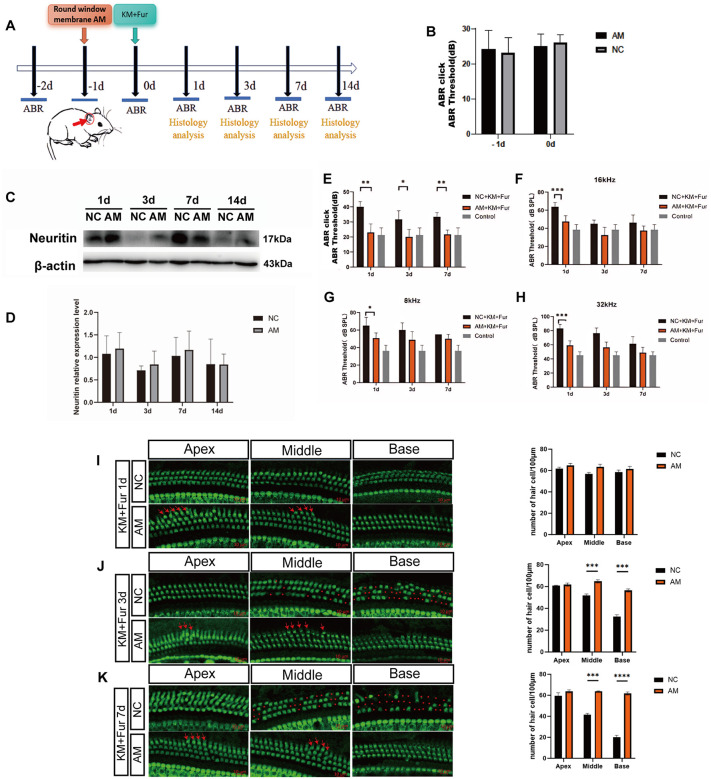
Target miRNA inhibitors improve auditory function in a mouse model of low-dose hearing loss. **A** Schematic diagram of the experimental design. **B** Statistical analysis of the changes in mouse hearing thresholds after drug damage (*p* < 0.05). **C-D** Western blot analysis of Neuritin expression in the cochlea of mice 1 day, 3 days, 7 days and 14 days after AMs intervention. **E-H** ABR thresholds were measured in response to click (**E**) and tone burst (**F-H**) stimuli at 1, 3, 7, and 14 days after AMs treatment. **I-K**.Immunofluorescence staining of cochlear hair cells at 1, 3, and 7 days after AMs intervention. Red arrows indicate increased hair cells, and red stars indicate missing cells.

To further investigate the effects of AMs targeting miR-339-5p, miR-1198-5p, and miR-224-5p on auditory function and cochlear HCs across varying severities of SNHL, we performed experiments following the protocol outlined in Fig 7A. First, the safety of drug delivery via the round window membrane was confirmed through ABR measurements, which revealed no significant alteration in baseline hearing thresholds in mice (Fig 7B).

Subsequently, we examined the changes in Neuritin expression following AMs intervention. Between 1 and 3 days post-intervention, Neuritin protein levels in the experimental group were significantly higher than those in the negative control (NC) group, indicating that the three AMs were successfully delivered into the cochlea via the round window membrane and effectively upregulated Neuritin expression. From day 7 to day 14, no significant difference in Neuritin expression was observed between the two groups (Fig 7C–D), a finding potentially attributable to the onset of increased endogenous Neuritin expression starting at day 7 after SNHL (Fig 1C).

**Fig 8:**
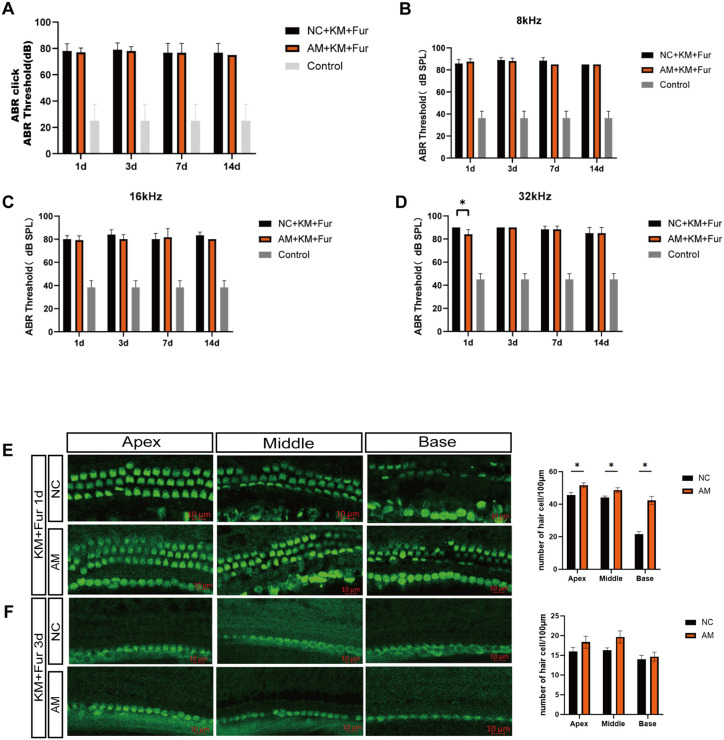
Effect of target miRNA inhibitors on auditory function in the high-dose mouse model. **A-D** ABR thresholds were measured in response to click (**A**) and tone burst (**B-D**) stimuli at 1, 3, 7, and 14 days after AMs treatment. **E-F** The immunofluorescence results of cochlear hair cells in mice after 1 day and 3 days of AMs intervention.

In the low-dose model, ABR measurements revealed that on day 1 post-modeling, the hearing threshold in the NC group increased by 10–20 dB, whereas the AMs group maintained thresholds within the normal range. From days 3–7, the NC group exhibited partial threshold recovery, but hearing in the AMs group remained consistently normal (Fig 8A). Tone burst ABR (tb-ABR) showed elevated thresholds in the NC group at 8, 16, and 32 kHz. Although the AMs group also displayed a threshold increase on day 1, the magnitude was 10–20 dB lower compared to the NC group. By days 3–7, thresholds in the NC group remained above normal levels, while those in the AMs group had largely recovered to near-normal values (Fig 8B–D).

The above results demonstrate that the AMs mixture significantly reduces the overall hearing threshold in mice with moderate hearing loss and enhances frequency-specific hearing across all cochlear turns (apical, middle, and basal turns), thereby promoting auditory functional recovery (Fig 7F–H).

Immunofluorescence staining of HCs revealed that in the NC group, on day 1, the arrangement of hair cells in the apical and middle turns was slightly disorganized, with no obvious loss observed in the basal turn. By day 3, minor HCs loss appeared in the middle turn, while loss in the basal turn became pronounced. By day 7, HCs loss across all cochlear turns had intensified, consistent with the typical progression of drug-induced hearing loss. In contrast, the AMs intervention group exhibited well-organized HCs arrays in the apical and middle turns on days 1, 3, and 7, along with an increased number of HCs (red arrows). In the basal turn, both HCs quantity and structural integrity were largely restored to near-normal levels (Fig 7I–K). Statistical analysis of cell counts demonstrated a significant intergroup difference, indicating that AMs treatment effectively attenuated HCs damage and promoted structural repair and numerical recovery.

However, in the high-dose model, HCs loss was profound, and AMs intervention failed to elicit significant improvements in hearing thresholds or HCs counts (Fig 8).

## Discussion

SNHL significantly affects the health and quality of life of patients and is associated with various conditions, such as mental disorders and Alzheimer’s disease [28,33]. Given that HCs are essential for auditory perception, maintaining their survival, function, and quantity represents a promising therapeutic approach for SNHL management [34]. As shown in our previous studies, Neuritin is significantly downregulated in SNHL, and recombinant Neuritin protein can promote the proliferation of hair cells [25,26]. Nevertheless, little is known about the molecular mechanism underlying the decreased expression of Neuritin.

A subclass of non-coding RNAs known as miRNAs suppresses gene expression at post-transcriptional levels [29,35]. There is growing evidence that miRNAs are involved in many physiological and pathological processes. Research indicate that miRNAs play a significant role in the inner ear, being indispensable not only during embryonic development, differentiation, and maturation but also for hair cells survival after birth [27]. As research progresses, an increasing number of miRNAs have been identified in the inner ear. For example, the mouse inner ear expresses about one-third of all known miRNAs [36], indicating that they may work together to regulate biological processes relevant to hearing. In this study, RNA-seq was employed to profile miRNA expression in the Organ of Corti of a mousee model of SNHL, leading to the identification of 24 upregulated miRNAs. Bioinformatic target prediction analysis revealed that 15 of these miRNAs may directly target the Neuritin gene, implicating them in the pathogenesis of hearing loss through modulation of Neuritin expression.

We initially used qRT-PCR to verify the above bioinformatics predictions and found that 12 out of 15 candidate miRNAs were markedly elevated in the Organ of Corti of the hearing loss mouse model. A total of nine miRNAs were chosen for functional validation based on their expression levels and detection reliability (18 < Ct < 30), considering the low quantity of miRNAs in tissues. Based on the canonical mechanism of miRNA-mediated gene silencing via the 3′-UTR [37], synthetic miRNA mimics were transfected into 293T cells. Western blot analysis revealed that miR-145a-5p, miR-224-5p, miR-339-5p, and miR-1198-5p significantly suppressed Neuritin protein expression. The direct binding of miR-224-5p, miR-339-5p, and miR-1198-5p to the Neuritin 3′-UTR was validated by subsequent luciferase reporter experiments; however, miR-145a-5p did not exhibit effective binding, possibly due to the absence of adequate binding sites or limitations in prediction accuracy. Therefore, we concluded that the decreased expression of Neuritin in SNHL is caused by targeted inhibition by miR-224-5p, miR-339-5p, and miR-1198-5p.

MiRNAs have been found to be associated with the occurrence and development of various human diseases [38]. Therefore, miRNAs are currently the focus of research as potential diagnostic predictors or biomarkers and therapeutic targets for various diseases, such as cancer [37], heart disease [39], diabetes [40], and inner ear cell injury [27]. Evidence indicates that increasing the number of targeted miRNAs from one to two and then to three results in a cumulative increase in HCs number, suggesting a dose-dependent and additive effect of miRNA regulation on HC survival [41]. In this study, following gentamicin-induced damage, cultured basilar membranes were co-treated in vitro with inhibitors targeting miR-224-5p, miR-339-5p, and miR-1198-5p mixed at an equimolar ratio (1:1:1). The findings showed that the group treated with the inhibitor mixture had more HCs arranged in a well-organized manner, suggesting that concurrent inhibition of miR-224-5p, miR-339-5p, and miR-1198-5p protects the Organ of Corti from gentamicin-induced damage to HCs.

To further explore the effects of miR-224-5p, miR-339-5p and miR-1198-5p AMs on the auditory function of mice with hearing loss, we injected a mixture of the AMs into the round window of the mouse cochlea and assessed auditory function using tone burst auditory brainstem responses (tb-ABR). The tb-ABR measurements at 8, 16, and 32 kHz reflect the functional status of the apical, middle, and basal turns, respectively [42]. However, the frequency specificity of tb-ABR is not absolute; low-frequency stimulation can activate broader cochlear regions, and the ABR threshold represents the summed response of a limited cochlear region rather than that of a discrete point [43]. Using the low-dose hearing loss model, AMs therapy promoted the recovery of HC counts in the middle and apical turns and significantly improved auditory thresholds. On the other hand, the high-dose SNHL model did not show any discernible improvement in hearing function. Researches show that aminoglycoside medications can produce excessive reactive oxygen species (ROS) and trigger inflammation, which can damage HCs permanently [44]. Additionally, 20% to 47% of patients may experience irreversible hearing loss [2,45]. Moreover, due to the terminal differentiation, it is challenging for hair cells to recover following damage or degeneration [46]. Therefore, identifying an external intervention to promote HCs regeneration or repair is of great important from a research perspective. According to our earlier research, Neuritin can promote the growth of SC progenitor cells and transdifferentiation of supporting cells into HCs [25]. According to the study’s findings, AMs may promote HCs survival and regeneration by relieving the inhibition of Neuritin and triggering subsequent repair processes. However, in cases of severe injury, the loss of HCs might outweigh the ability of Neuritin to repair them, which would mean that AMs therapy wouldn’t have any meaningful therapeutic effects. More research is necessary to determine the exact mechanism by which Neuritin supports HCs survival and regeneration.

In conclusion, this study elucidates the molecular mechanism by which Neuritin is downregulated in SNHL, namely targeted inhibition by miR-224-5p, miR-339-5p, and miR-1198-5p. Notably, the therapeutic efficacy of AMs was model-dependent: in the low-dose model, AMs treatment significantly promoted hair cell recovery in the middle and apical turns and markedly enhanced auditory thresholds; however, in the high-dose model, no discernible improvement in hearing function was observed. These contrasting findings suggest that the protective effects of AMs may be limited to less severe injury, while more severe injury may require additional strategies. Nevertheless, the efficacy observed in the low-dose model highlights the potential of AMs as a therapeutic agent for hearing loss.

Nevertheless, this study has several limitations. First, a mixture of three miRNAs was used in the experiments, and the specific contribution of each individual miRNA to the overall effect requires further experimental validation. Second, although an increase in the number of hair cells was observed in the mouse cochlea following AM intervention, the origin of these newly increased hair cells remains unclear and warrants further investigation. Additionally, we have not systematically evaluated the off-target effects of these three miRNA inhibitors; therefore, the possibility of their impact on non-target genes cannot be completely excluded. These issues will be addressed in future studies.

## Supporting information

S1 File Raw images of all blots and gels. This PDF file contains the original, uncropped images for all figures reporting blot/gel results, with annotations for sample identity, loading order, molecular weight markers, and corresponding figure panels.
(PDF)
