# Supplementary material for: The inhibitors of miR-224-5p, miR-339-5p, and miR-1198-5p improve auditory function by promoting the expression of neuritin in hearing loss mice
Source: PLoS One. 2026 May 21;21(5):e0349821. doi: 10.1371/journal.pone.0349821 (PMC13193335; doi:10.1371/journal.pone.0349821)

Fig 1C

Blot:Neuritin

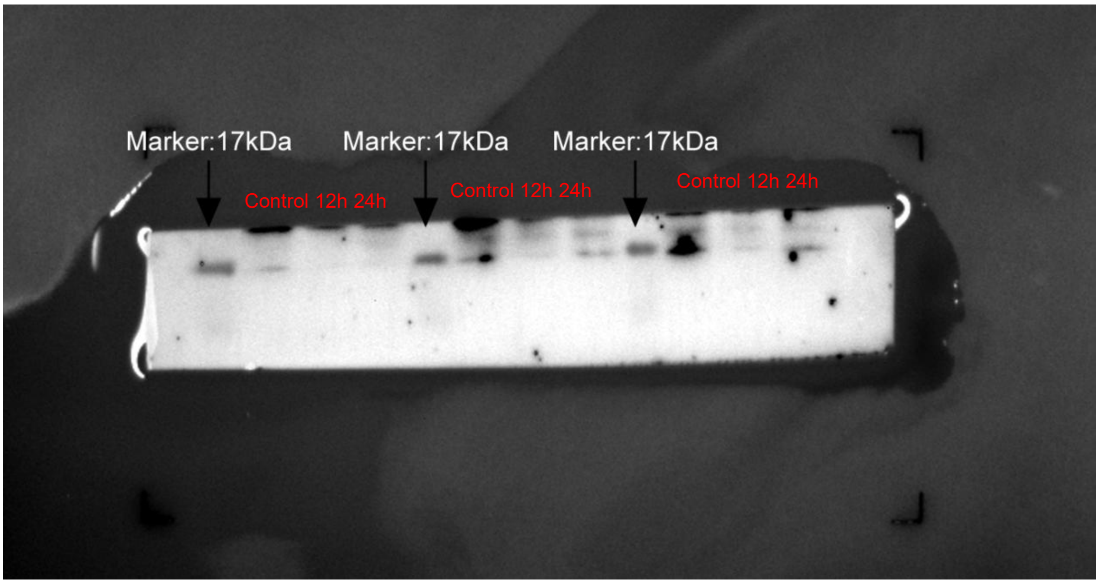

Blot: β-actin

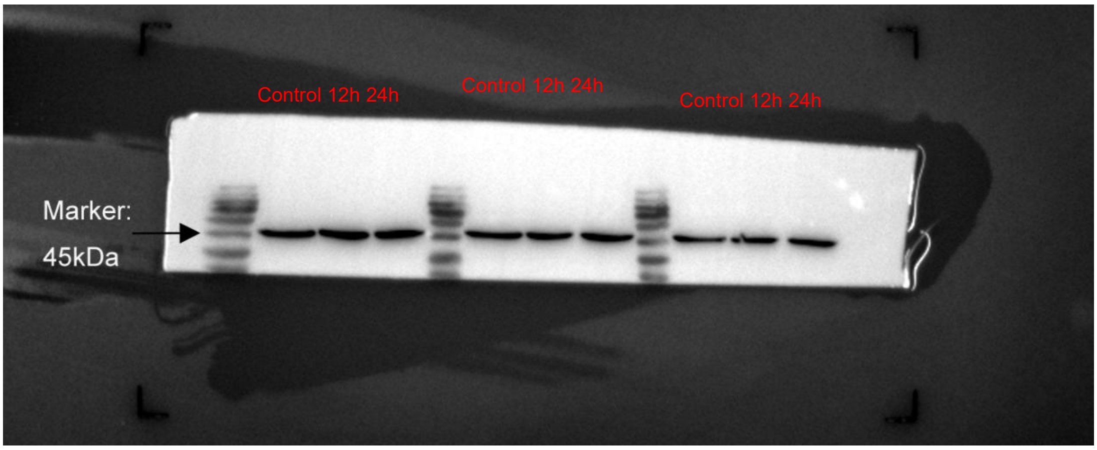

Fig 1D

Blot:Neuritin

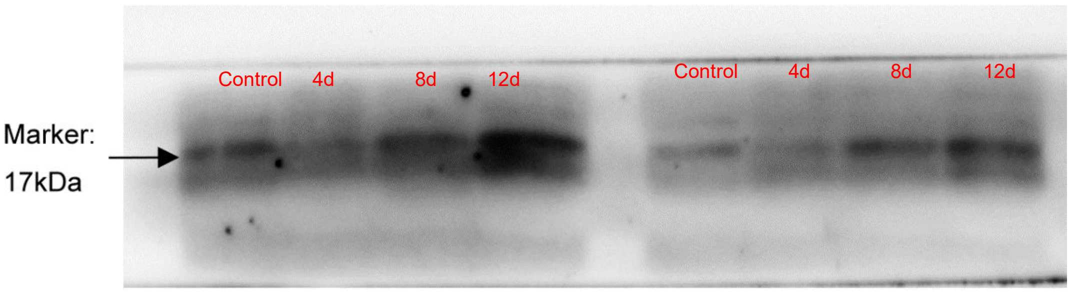

Blot: β-actin

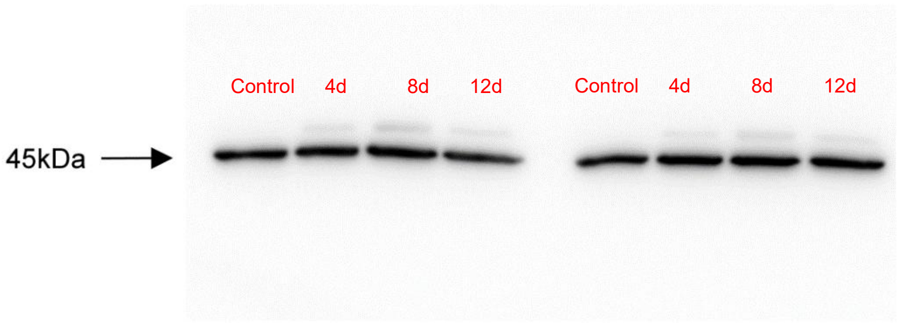

Fig 4A（miR-145a-5p）

Blot:Neuritin

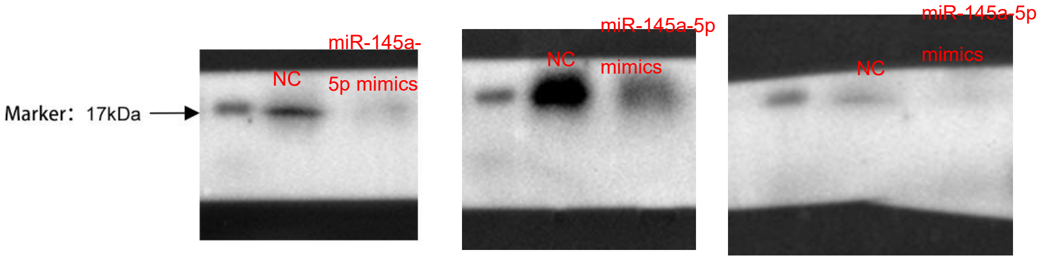

Blot: β-actin

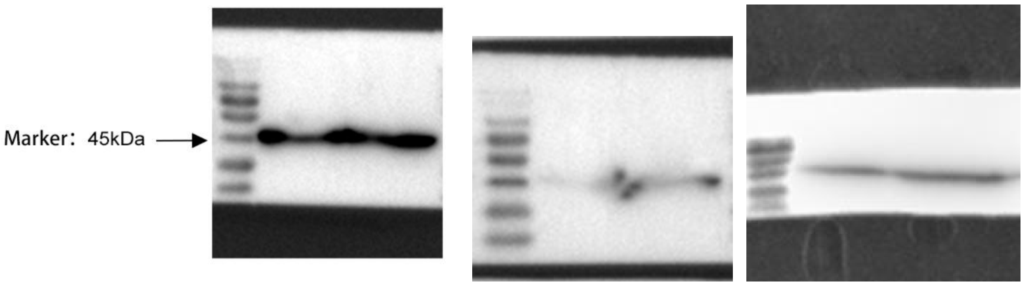

Fig 4B（miR-224-5p）

Blot:Neuritin

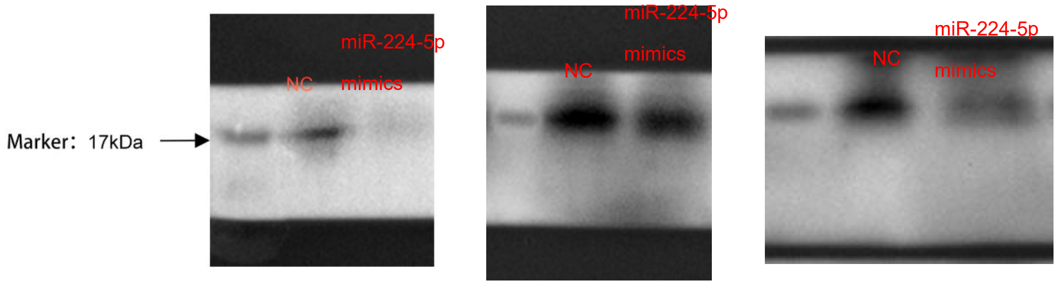

Blot： β-actin

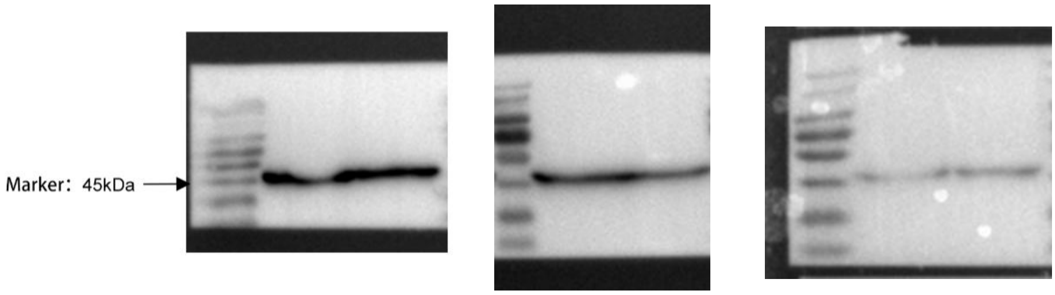

Fig 4C （miR-339-5p）

Blot:Neuritin

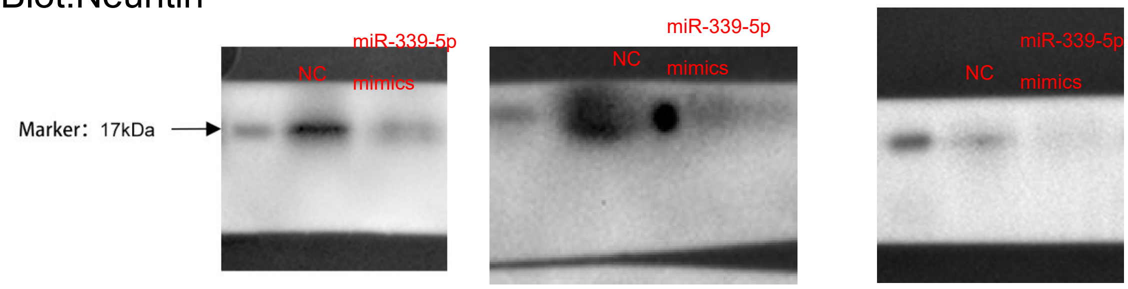

Blot： β-actin

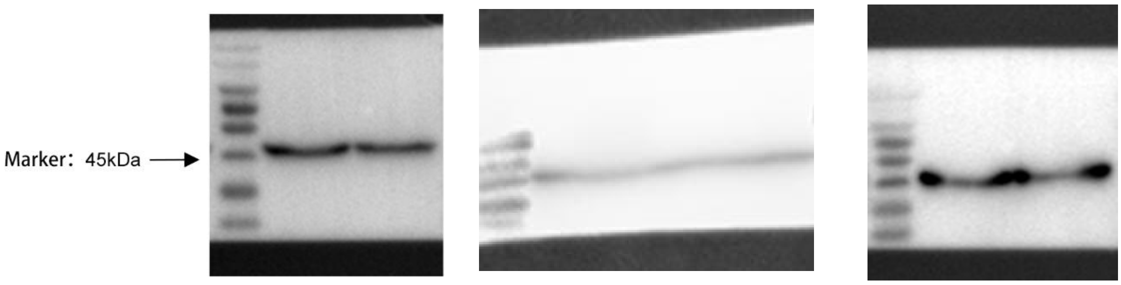

Fig 4D（miR-1198-5p）

Blot:Neuritin

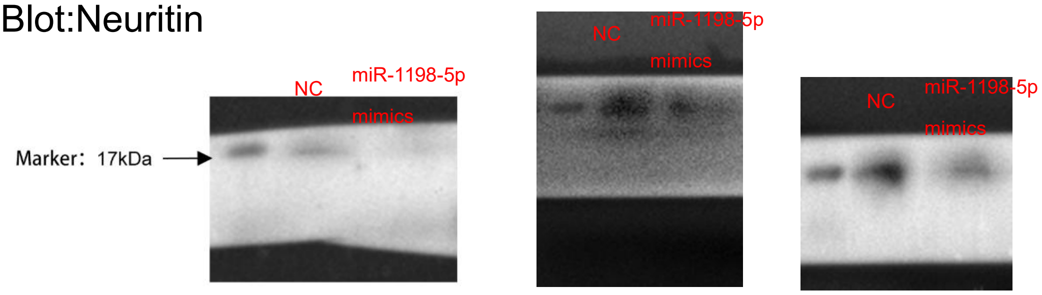

Blot： β-actin

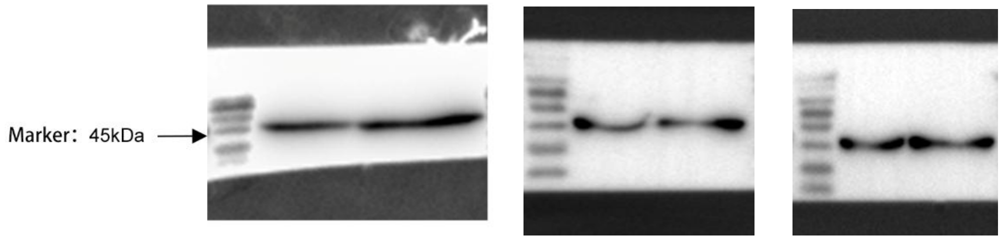

Fig 4E（miR-93-3p）

Blot:Neuritin

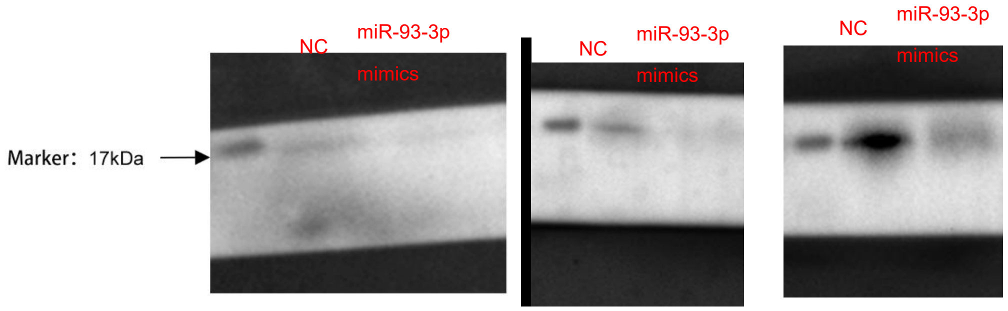

Blot： β-actin

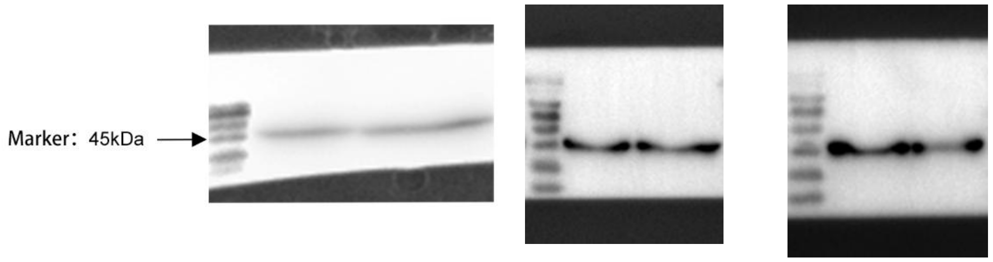

Fig 4F（miR-181a-2-3p）

Blot:Neuritin

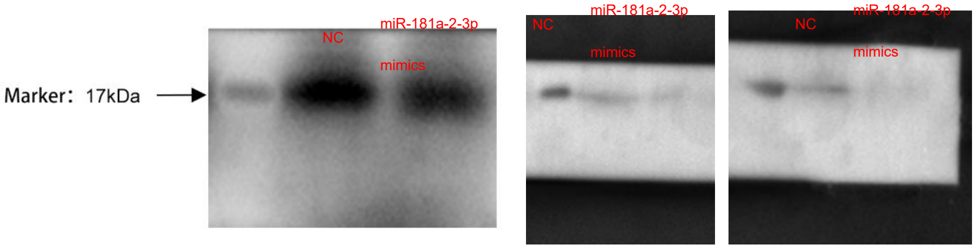

Blot： β-actin

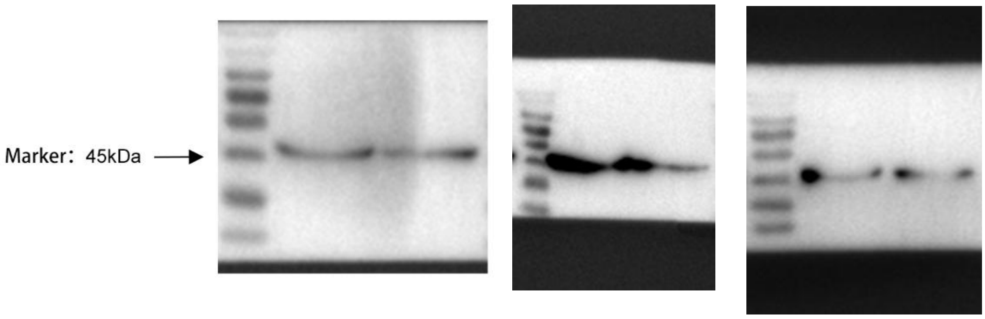

Fig 4G（miR-21a-3p）

Blot:Neuritin

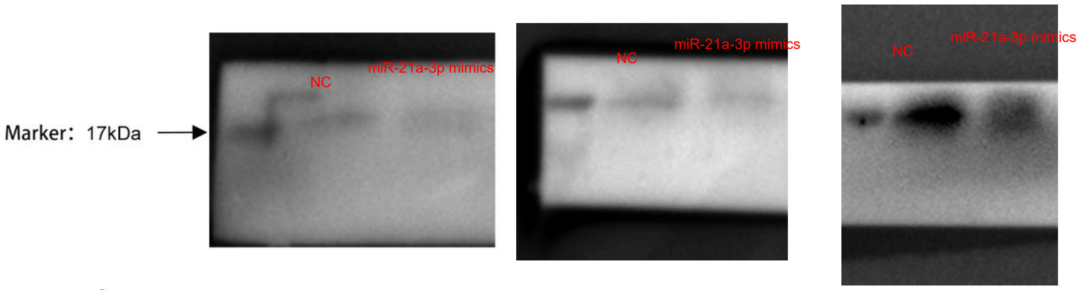

Blot： β-actin

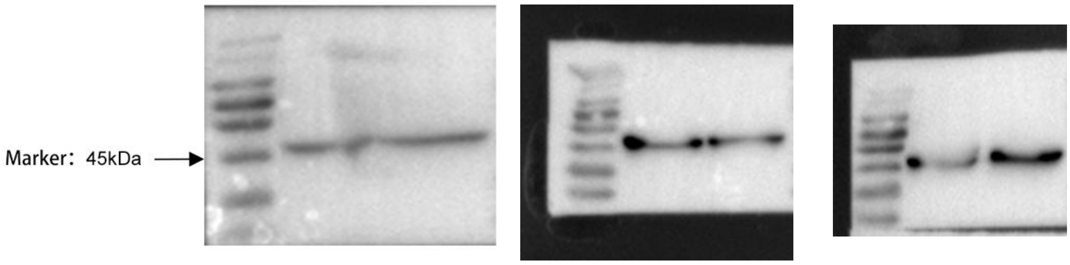

Fig 4H（miR-1247-3p）

Blot:Neuritin

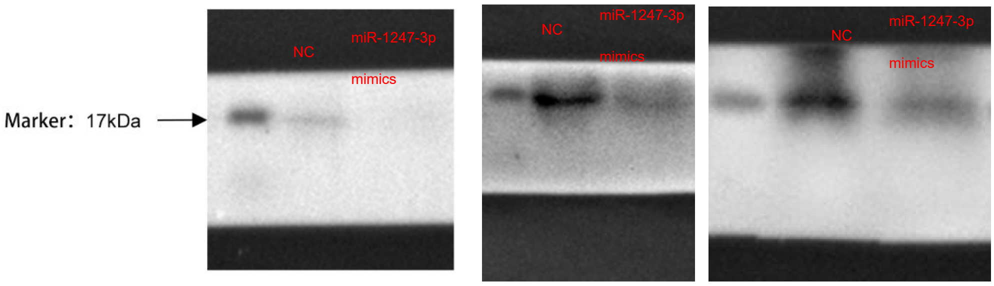

Blot：β-actin

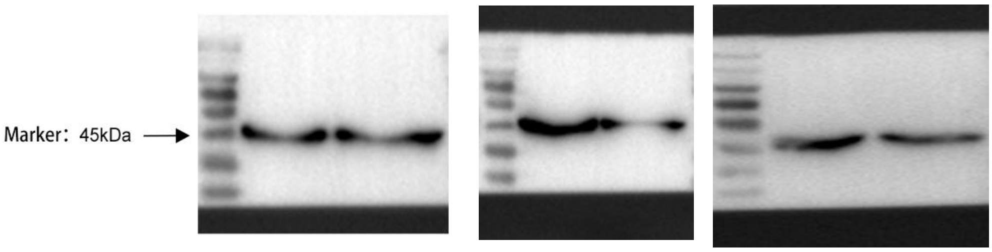

Fig 4I（miR-1247-5p）

Blot:Neuritin

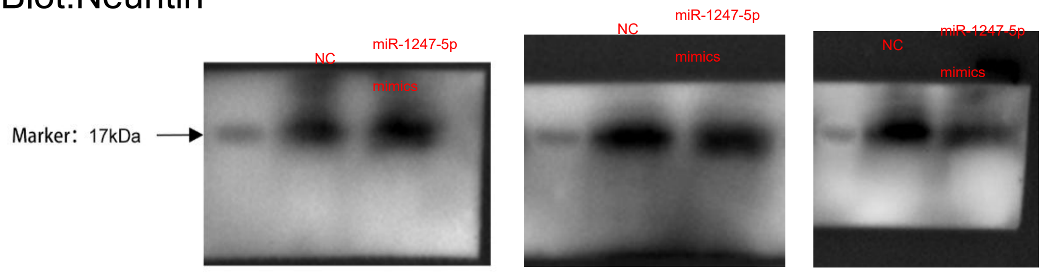

Blot：β-actin

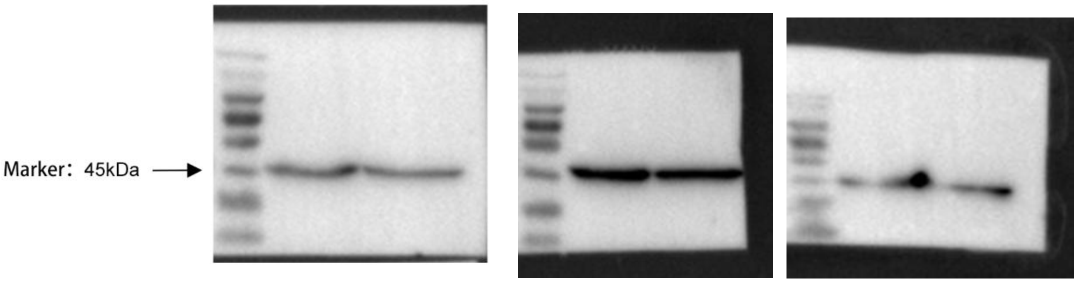

Fig 7C:

Blot:Neuritin

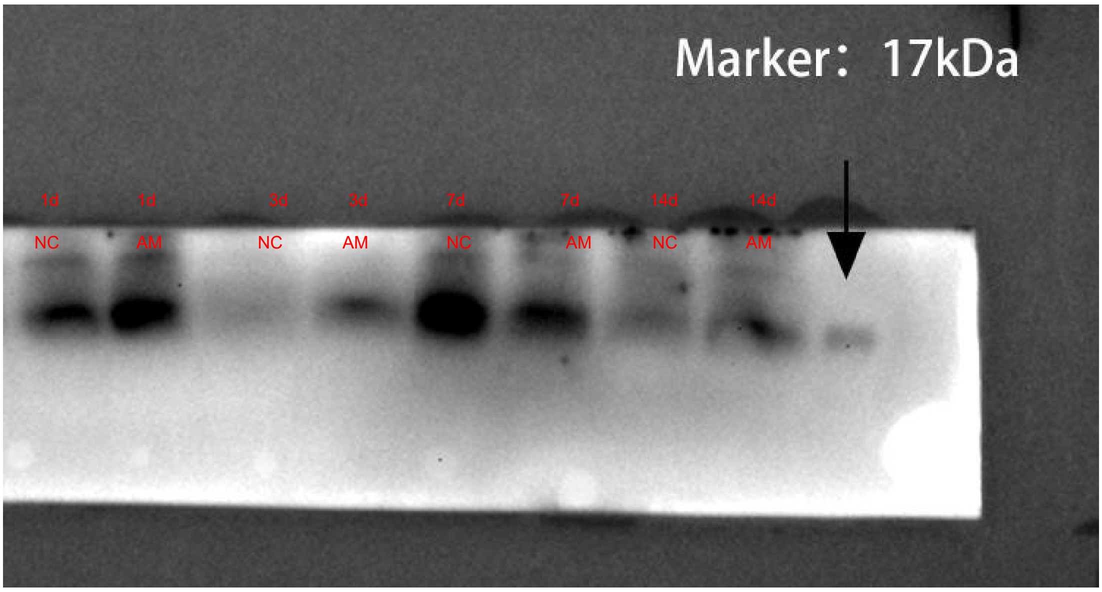

Blot： β-actin

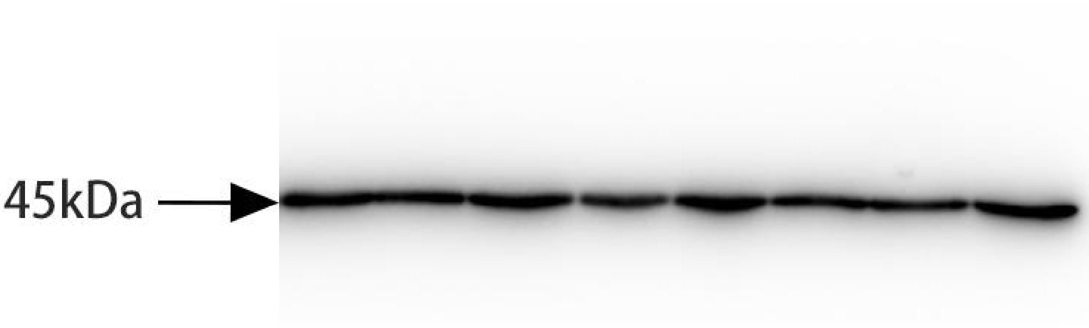

Supplement: S1 File — This PDF file contains the original, uncropped images for all figures reporting blot/gel results, with annotations for sample identity, loading order, molecular weight markers, and corresponding figure panels. (PDF) [file pone.0349821.s001.pdf]
